# What can veterinary professionals do? Measuring the effect of one domestic violence training pilot program on veterinary professionals' capacity to recognize, respond, and refer human victims of domestic violence

**DOI:** 10.3389/fvets.2024.1254373

**Published:** 2024-02-13

**Authors:** Rochelle Paterson, Elise Boller, Youna Kim, Kate Hammond, Kristin Diemer

**Affiliations:** ^1^Faculty of Veterinary and Agricultural Sciences, University of Melbourne, Melbourne, VIC, Australia; ^2^Eastern Domestic Violence Service (EDVOS), Melbourne, VIC, Australia; ^3^Faculty of Medicine, Dentistry and Health Sciences, University of Melbourne, Melbourne, VIC, Australia

**Keywords:** domestic violence, animal abuse, training, veterinary, pet abuse, the link

## Abstract

**Introduction:**

Veterinary professionals have a key role in facilitating multi-agency collaboration to prevent and respond to domestic violence (DV) in situations where animals may be directly or indirectly involved. Yet despite their position as potential touchpoints for victim-survivors with animals, many veterinary professionals do not feel equipped to act on suspicions or disclosures of DV. In response to this identified need, one service operating in Melbourne, Australia, developed the Vet-3R's training program (Recognize-Respond-Refer) which was piloted on 65 veterinary professionals in Melbourne's Eastern Metropolitan Region.

**Methods:**

This is an exploratory study aimed at measuring the effect of the Vets 3-R's program on veterinary professionals' confidence and capacity to recognize, respond and refer victims of DV. Participants were invited to complete online surveys before and after the training to evaluate their understanding of DV and their capacity to support suspected victim-survivors with animals who present at their service.

**Results:**

The pre-training self-evaluation scores indicated that while most veterinary professionals are aware of the link between animal abuse and DV, they lack the confidence to respond and refer individuals when confronted with suspicions or disclosures of abuse. However, upon completion of the Vets 3-R's program, participants reported marked improvements in their ability to recognize, respond, and refer victim-survivors. The most significant improvement could be seen in participants' self-reported ability to respond appropriately to suspicions of DV.

**Discussion:**

While results are indicative only due to the small sample size, this study suggests that veterinary professionals may be an underutilized intervention point for DV victim-survivors with animals. The Vet-3R's training program can be a useful tool for increasing effectiveness of this intervention point to safely assist DV victim-survivors. More research on similar programs with a larger cohort of participants would be beneficial to measure the impact of such programs on a wider scale.

## 1 Introduction

### 1.1 The link between pet abuse and domestic violence

In recent years an increasing amount of research has been dedicated to exposing the link between pet abuse and domestic violence (DV). As proposed by Arkow in 1996, “when animals are abused, people are at risk; when people are abused, animals are at risk” (1). This statement succinctly expresses decades of psychosocial research into the link of pet abuse and human violence. In particular, how the orchestrated harm of a pet is instrumentalized by abusers to purposefully threaten a human victim. Companion animal abuse aims to demonstrate power, violate trust, force compliance, and punish those who love the pet. Ultimately, it creates a culture of normalized violence and provides the perpetrator with a sense of control over both their human and animal victims (2).

Research has repeatedly demonstrated strong links between animal abuse and DV (3–13). One study by Arkow, for instance, showed that in 71% of respondents experiencing DV, the perpetrator was concurrently abusing or neglecting a pet. This was compared to 6% in households not experiencing DV (4). Domestic violence and animal abuse frequently co-occurs, and the existence of threats or harm toward animals within a home may be an indication that harm toward humans is occurring (3, 14). The abuse directed toward animals often includes threats of violence, actual violence, killing the animal, or threatening to give the animal away (15, 16).

In addition to animal abuse being a predictor for human abuse, it may also be a risk factor for severe domestic violence, as abusers who are cruel to animals demonstrate higher rates of sexual violence and use more controlling behaviors including isolation, intimidation, threats, and economic abuse (9). Women reporting abuse by an intimate partner with a history of animal mistreatment are more likely to report being strangled (79%) and being forced to have sex (26%), underscoring the extremely high-risk environments to which victims are exposed (17).

Research on perpetrators of both DV and animal abuse also finds a strong correlation between the two offenses. In 2008, for instance, Gullone and Clarke found that 55% of individuals arrested for animal abuse in the Australian state of New South Wales had a previous record of DV, while a further 17% had records of committing sexual assault (18). This is similar to 2014 study of 207 men referred to a Batterer Intervention Program in Rhode Island (USA), which found that 41% of men arrested for DV admitted to committing animal cruelty as adults (19).

This research on the link between violence to humans and violence toward animals, particularly in the context of DV, demonstrates an opportunity for the presence of animals or animal abuse to be utilized more consistently across various agencies as an opportunity for intervention. As such, the interface between veterinary professionals and their clients may be an opportunity for awareness and intervention. This study aimed to deepen understanding of how a training program might affect veterinary professionals' capacity and willingness to recognize, respond and refer DV victims.

### 1.2 Challenges associated with the presence of an animal in a DV situation

A pet is often one of the few valued sources of trust and companionship for a victim of DV, especially when they have been isolated from their friends, family, and community as a deliberate tactic of abuse. Indeed, pets and animals that are well cared for have long been seen as protective factors for human health (2), and have consequently been incorporated into human therapies and interventions through programs such as therapeutic horseback riding, disability support animals, and even dogs in prisons. In 2000, Flynn described how victims of interpersonal violence (IPV) often regard their pets as not just family members but as children (20). The relationship with one's pet is seen as consistent and secure (21). Thus, not only can animal abuse be an important sentinel for DV, but the roles that pets play in the lives of abused people must be taken seriously and, ideally be protected (22).

As such, safety planning and concern for a pet can make the decision to leave to an abusive household even more complicated, with a reported range of 20–48% of women delaying leaving an abusive situation out of fear for their animal's welfare (3, 7, 23). Safety planning is already a difficult and often non-linear process, and the existence of an animal in a DV situation can create additional roadblocks to safety for both human and animal victim-survivors (24). In the first instance, if the pet does leave with the victim, the options for accommodation are considerably limited. In a 2012 survey of 767 DV shelter workers across the US, 77.2% responded affirmatively when asked if animal abuse in a violent relationship led to greater fear and reduced the likelihood of seeking help. Despite this, only 44.6% reported their intake interview included questions concerning pet abuse and only 6% allowed pets to stay on-site at the shelter (25). This low percentage is again reflected in a later, 2018 study by Gray, who reported that out of 337 first-stage DV shelters in Canada, only 1% describe offering on-site pet programs (26). If accommodation does happen to be pet friendly, there may be barriers in terms of access, such as limitations on the length of stay (27). This can leave vulnerable people who have companion animals with few, or no, options when seeking to leave violent situations together. With DV being a pathway to homelessness for women—and in Australia, the leading cause of homelessness for women (28)—the inaccessibility of animal-friendly accommodation is likely contributing to this crisis (29).

Fortunately, in recent years, increased focus has been given to creating animal-friendly DV accommodation, with international initiatives such as Sheltering Animals & Families Together (SAF-T) providing guidance to shelters and refuges on how to house families with their pets (30). Additionally, many animal organizations have implemented foster care programs for pets whose carers are leaving DV situations and cannot access animal-friendly accommodation (31–34). Although housing animals together with their humans is considered best practice, these programs provide an additional avenue for human and animal safety.

Within the DV professional community, there has been increasing focus on addressing the needs of clients with pets. Eastern Domestic Violence Services (EDVOS)^1^ was one such service advocating for and incorporating animal-inclusive practices into domestic violence support services. EDVOS, operating in Melbourne, Australia, launched the Vets Against Violence project in 2018 with the view that veterinarians have the capacity to recognize, respond and refer victims of DV in a safe manner if appropriately trained. Indeed, many argue that education is crucial to prepare veterinarians for their response to pet abuse and DV in practice (35). Loss of self-esteem and self-confidence are among the many psychological impacts of DV (36). EDVOS therefore developed their Vet-3R's training program (Recognize-Respond-Refer) to prepare veterinary professionals to open conversations with their clients and guide suspected victims toward specialist support.

### 1.3 The role of veterinary professionals in DV prevention and response

Domestic violence is a significant and persistent problem across all societies, and veterinarians' interactions with citizens present a unique opportunity to create robust intervention points for victim-survivors with animals. Intimate partner violence is one of the most common forms of violence against women, with the World Health Organization estimating that 26 per cent of women who have ever been in a relationship with a partner globally have experienced physical and/or sexual violence by an intimate partner in their lifetime (37). In Australia, prevalence data shows that one in three Australian heterosexual women have experienced violence from a male partner (38), and DV is the leading preventable contributor to death, disability and illness in Australian women aged 18–44 (39). On average, Australian police deal with a DV incident every 2 min (40), a figure which does not capture the many cases of DV which remain unreported (41). In relation to the 2016 report from the Royal Commission into Family Violence in the Australian state of Victoria, the Premier labeled DV “the most urgent law and order emergency occurring in our state and the most unspeakable crime unfolding across our nation” (42).

Despite this sense of urgency, there still exists a large degree of stigma surrounding DV, both globally and in Australia (37, 43). Reports from the 2017 Australian National Community Attitudes toward violence against women Survey (NCAS) highlighted the prevalence of prejudicial attitudes among the community. Although declining, a small proportion of Australians (12%) still agree that DV is a private family matter, while 1 in 3 Australians still believe that if a woman does not leave her abusive partner, then she is responsible for continued violence. There also appears to be mistrust of women's reports of violence, with 23% of respondents to the NCAS agreeing that women exaggerate problems of male violence (44).

With such high rates of DV, and considering the immeasurable damage done to victim-survivors, families, and the wider community, incorporating innovative practices into DV prevention and response is greatly needed. Veterinary practices are community-based services that frequently interact with victim-survivors, but are infrequently utilized in DV response. Recently, members of the veterinary profession have been identified as potential respondents to assist individuals and their pets in crisis situations, and research has increasingly shown that vets have a role to play in the nexus of DV prevention and response (45–47). With animal ownership, DV, and animal abuse as a form of DV being common across the globe (3–13, 37, 48–50), the likelihood that a veterinarian (or other veterinary professional) will come into contact with both humans and animals experiencing abuse is, by definition, likely to be high. A 2005 survey by Green and Gullone, for instance, reported that 91% of Australian veterinarians have experienced cases of animal abuse in practice and almost a quarter reported known or suspected human abuse (51).

Despite this figure, studies have portrayed the veterinary community as being both uncertain and underprepared to recognize, respond and refer human victims of DV (35, 45, 52). One systematic review of global studies on veterinarian's role in responding to DV involving animals demonstrated that although between 42.8% and 86% of veterinarians are aware of the link between DV and animal abuse, most are not being trained to intervene in cases of animal abuse and human violence and are consequently not doing so (45). A 2018 thematic literature review by Newland listed the factors contributing to this inaction: in addition to the lack of training in this area, factors included a lack of knowledge of appropriate actions or of relevant services to contact, lack of knowledge of ethical and legal obligations in regards to client and patient confidentiality, and lack of available time to adequately discuss concerns (35). There is also still the commonly cited fear of venturing into a subject traditionally seen as private (51).

Professionals in the community agree that veterinary education has typically included inadequate information about abuse identification and prevention (53). Despite the existing research on features of non-accidental injury to animals—such as associated fracture and injury patterns and case attributes—application of this research in the field is low (46, 47, 54, 55). In 1999, Landau reported that among 31 veterinary schools across North America, students received an average of 76 min of associated training regarding pet abuse and 8 min pertaining to DV (56). Younger veterinarians have moreover been shown to disagree when asked if they received adequate training on this subject (51). Despite this, there is a strong interest in changing this figure, with a 2017 survey of 1,155 veterinarians reporting that 72.7% were interested in receiving further education on the topic (57). In response to this need for education, organizations such as The Link Group in the United Kingdom, and Lucy's Project in Australia have provided training and educational resources to veterinary professionals (58) and veterinary students (59). However, widespread engagement and education of veterinary professionals to become an intervention point for DV is yet to be achieved.

## 2 Materials and methods

The present research is an exploratory study aimed to determine the baseline understanding of a small sample of Melbourne-based veterinary professionals' knowledge and understanding of the link between animal abuse and DV, and their capacity to recognize, respond and refer human victims. It then aimed to measure the impact of the Vet-3R's training program to determine whether the program effected any changes on participants' confidence and capacity to support DV victim-survivors. Human Ethics Approval for the study was granted by the University of Melbourne office of research ethics and integrity.

### 2.1 The Vet-3R's training program

The Vet-3R's training consisted of a 2.5-h face-to-face program with slide presentation and informal discussion, held at a venue within the Boroondara city council region. Five training sessions were held in total, each organized and provided by EDVOS. The training encouraged participants to critically challenge the commonly held myths associated with DV and addressed its gendered nature. It presented key facts regarding the strength of the link between animal abuse and DV before communicating how to recognize DV, and how veterinary professionals can safely and appropriately respond and refer human victims.

### 2.2 Data collection

Veterinary hospitals, veterinary teaching facilities and animal management offices in Melbourne were contacted and recruited for participation in the Vet-3R's training by EDVOS. Responding veterinary professionals (veterinarians, nurses, students, administrative staff, and animal management officers) registered for one of five training sessions. Registered attendees were then contacted independently by email and invited to participate in the study. Study participation involved completion of a short, 5–10 min survey both before and after the training session through the secure web platform REDCap (Research Electronic Data Capture). Entering the survey formed implied consent after having displayed the Plain Language Statement.

The pre-training survey included questions about demographic characteristics of the participant before asking participants to self-evaluate on six different statements. Patients responded via a slider scale marked 0 to 100 whereby 0 indicated a low understanding, 50 indicated some understanding and 100 indicated a high understanding.

Following the self-evaluation questions, participants were presented with several “calibrating” questions to measure their understanding of issues related to animal abuse and DV. These questions were included to act as an additional measurement of the effectiveness of the training in increasing participants' knowledge on the topic, as well as a form of bias-testing—i.e., to compare participants' true level understanding with their self-identified level of understanding. In doing so, these questions were able to highlight any discrepancies caused by issues such as Reference Group Effect and other biases that may impact people's ability to accurately self-evaluate their level of knowledge (60, 61).

The post-training survey was identical to the pre-training survey except that demographic information was not again collected. There were no designated control groups in this evaluative study. Rather, the study was designed so that participants acted in their own controls in that they completed the survey both before and after the intervention (Vet-3R's training).

### 2.3 Participants' demographic data

Of the 65 registered attendees of the Vet-3R's training, 39 participants (60%) agreed to be involved in the study. Participant responses in relation to gender identity, age and years' experience as a veterinary professional are presented in Table 1.

**Table 1:** Demographic characteristics of participants.

	**N**	**(%)**
**Gender identity**		
Female	33	(84.6)
Male	5	(12.8)
Other/Unspecified	1	(2.6)
**Age group (years)**		
18–25	6	(15.4)
26–35	13	(33.3)
36–45	9	(23.1)
46–55	9	(23.1)
56+	2	(5.1)
**Industry experience (years)**		
<5	19	(50.0)
5–10	6	(15.8)
11–20	8	(21.1)
21–30	3	(7.9)
31+	2	(5.3)
**Documented involvement in domestic violence programs**		
Yes	10	(25.6)
No	29	(74.4)
**Experienced personal threats of domestic violence**		
Yes	12	(30.8)
No	27	(69.2)

Participants were also asked whether they had ever been involved in DV issues (e.g., documented involvement in advocacy work with victims of DV). Participants who selected “yes” were given the opportunity to describe their involvement. Responses to this question included donating household items to charities working with victims of DV, to assisting an individual or their pet who has been affected by DV, to being directly involved in advocacy work and to being a sworn police officer in a previous career. Some participants also had lived experience of DV.

## 3 Results

### 3.1 Participant self-evaluation of their understanding of DV involving animals

In both the pre-training and post-training surveys, participants were asked to self-evaluate on six different statements as a reflection of their confidence and capacity to recognize, respond and refer potential or actual victims of DV. Participants did this through marking on a scale of 1–100 their level of understanding or ability related to the following six statements, with 0 indicating low understanding or ability, and 100 indicating high understanding or ability:

1. “My understanding of the strength of the link between animal abuse and domestic violence;”
2. “My understanding of the gendered nature of domestic violence;”
3. “My ability to recognize signs of domestic violence;”
4. “My capacity to respond appropriately if I suspect domestic violence;”
5. “My capacity to respond appropriately to disclosures of domestic violence;” and
6. “My capacity to refer appropriately, following disclosures of domestic violence.”

A total of 37 participants either completed or partially completed the pre-training survey. Of these 37 participants, 17 proceeded to complete the post-training survey after attending the Vet-3R's training, while an additional 2 participants completed the post-training survey without completing the pre-training survey beforehand. A total of 19 responses to the post-training survey were therefore recorded.

#### 3.1.1 Statement 1: “My understanding of the strength of the link between animal abuse and domestic violence”

When asked to quantify “My understanding of the strength of the link between animal abuse and domestic violence,” the median response in the pre-training survey was 64.50 (*n* = 36). Participants in the 56+ age group, participants with 31+ years industry experience and participants with documented involvement in DV programs recorded the highest median pre-training self-evaluation scores for this statement, with 72.00, 85.00, and 70.00 respectively. Following the Vet-3R's training, the median response in the post-training survey was 85.00 (*n* = 19). Thus, there was an overall increase in the median self-evaluation score of the cohort by 20.50 units or by 31.78%, with 32.58% (*n* = 6/19) participants responding with the highest possible score of 100.00. Self-evaluation scores according to individual participant demographic can be seen in Table 2.

**Table 2:** Self-evaluation scores for the statement “My understanding of the strength of the link between animal abuse and domestic violence.”

	**Pre-training**			**Post-training**		
	**n**	**Median**	**Mean**	**n**	**Median**	**Mean**
Total participants	36	64.50	59.36	19	85.00	87.00
**Gender identity**						
Female	33	65.00	60.70	14	88.00	87.64
Male	2	35.00	35.00	4	86.50	88.25
Other/ unspecified	1	64.00	64.00	1	73.00	73.00
**Age group (years)**						
18–25	6	69.00	63.00	3	91.00	90.33
26–35	13	64.00	57.15	3	80.00	79.33
36–45	9	50.00	53.11	5	85.00	85.00
46–55	7	65.00	66.57	7	100.00	91.00
56+	1	72.00	72.00	1	82.00	82.00
**Industry experience (years)**						
<5	18	65.50	61.56	7	91.00	90.86
5–10	6	50.00	59.83	3	73.00	77.00
11–20	7	50.00	44.00	5	85.00	88.00
21–30	3	65.00	64.00	3	100.00	88.00
31+	2	85.00	85.00	1	82.00	82.00
**Documented involvement in domestic violence programs**						
Yes	10	70.00	70.50	5	88.00	89.20
No	26	50.00	55.08	14	85.00	86.21
**Experienced personal threats of domestic violence**						
Yes	11	69.00	69.09	7	85.00	86.29
No	25	50.00	55.08	12	88.00	87.42

#### 3.1.2 Statement 2: “My understanding of the gendered nature of domestic violence”

When asked to quantify “My understanding of the gendered nature of domestic violence,” the median response in the pre-training survey was 65.00 (*n* = 37), making it the highest median score of all statements. Following the Vet-3R's training, the median response in the post-training survey was 91.00 (*n* = 19), again the highest of all statements. There was an overall increase in the median self-evaluation score of the by 26.0 units or by 40.00%. The highest possible score of 100.00 was also recorded by 42.10% (*n* = 8/19) of participants.

Of note, there was a significant difference in the median pre-training score between veterinary professionals who had experienced personal threats of DV and those who responded that they had not. Those who had experienced personal threats recorded a median score of 79.00, compared to 51.00 in participants who had not experienced personal threats. However, following the Vet-3R's training, median scores between these two groups were similar at 88.00 and 91.50 respectively. Self-evaluation scores according to participant demographics can be seen in Table 3.

**Table 3:** Self-evaluation scores for the statement “My understanding of the gendered nature of domestic violence.”

	**Pre-training**			**Post-training**		
	**n**	**Median**	**Mean**	**n**	**Median**	**Mean**
Total participants	37	65.00	62.70	19	91.00	92.37
**Gender identity**						
Female	33	64.00	61.97	14	90.50	92.29
Male	3	90.00	70.00	4	100.00	97.00
Other/ unspecified	1	65.00	65.00	1	75.00	75.00
**Age group (years)**						
18–25	6	65.00	69.00	3	91.00	93.00
26–35	13	50.00	48.15	3	85.00	86.67
36–45	9	64.00	66.56	5	90.00	90.80
46–55	8	84.00	75.88	7	100.00	97.14
56+	1	74.00	74.00	1	82.00	82.00
**Industry experience (years)**						
<5	18	60.00	60.11	7	100.00	94.86
5–10	6	67.00	69.67	3	88.00	84.33
11–20	8	50.50	54.75	5	90.00	92.80
21–30	3	70.00	72.00	3	100.00	97.33
31+	2	83.00	83.00	1	82.00	82.00
**Documented involvement in domestic violence programs**						
Yes	10	79.00	70.80	5	88.00	91.60
No	27	60.00	56.37	14	91.50	92.64
**Experienced personal threats of domestic violence**						
Yes	12	79.00	80.75	7	88.00	90.43
No	25	51.00	54.04	12	91.50	93.50

#### 3.1.3 Statement 3: “My ability to recognize signs of domestic violence”

When asked to quantify “My ability to recognize signs of domestic violence,” the median response in the pre-training survey was 50.00 (*n* = 36), while the median response in the post-training survey was 77.00 (*n* = 19). Thus, there was an overall increase in the median self-evaluation score of the cohort by 27.00 units or by 54.00%. Self-evaluation scores according to participant demographics can be seen in Table 4.

**Table 4:** Self-evaluation scores for the statement “My ability to recognize signs of domestic violence.”

	**Pre-training**			**Post-training**		
	**n**	**Median**	**Mean**	**n**	**Median**	**Mean**
Total participants	36	50.00	49.47	19	77.00	81.21
**Gender identity**						
Female	33	50.00	49.39	14	74.50	79.86
Male	2	50.50	50.50	4	84.00	87.00
Other/ unspecified	1	50.00	50.00	1	77.00	77.00
**Age group (years)**						
18–25	6	50.00	50.33	3	82.00	81.00
26–35	13	50.00	42.15	3	77.00	75.67
36–45	9	50.00	51.00	5	77.00	82.40
46–55	7	70.00	56.29	7	80.00	83.86
56+	1	78.00	78.00	1	74.00	74.00
**Industry experience (years)**						
<5	18	50.00	46.50	7	82.00	84.71
5–10	6	50.00	59.50	3	77.00	78.33
11–20	7	50.00	41.14	5	80.00	84.40
21–30	3	50.00	50.33	3	74.00	73.00
31+	2	74.00	74.00	1	74.00	74.00
**Documented involvement in domestic violence programs**						
Yes	10	73.00	69.60	5	75.00	81.00
No	26	50.00	41.73	14	78.50	80.93
**Experienced personal threats of domestic violence**						
Yes	11	68.00	65.45	7	77.00	81.33
No	25	50.00	42.44	12	78.50	81.33

#### 3.1.4 Statement 4: “My capacity to respond appropriately if i suspect domestic violence”

When asked to quantify “My capacity to respond appropriately if I suspect domestic violence,” the median response in the pre-training survey was 44.50 (*n* = 36). This was the lowest self-evaluation score of all 6 statements. Particularly low scores were documented by the 18–25 years age group, by the <5 years industry experience group and by those who reported they had not experienced personal threats of DV.

The median response in the post-training survey was 80.00 (*n* = 19). Thus, there was an overall increase in the median self-evaluation score of the cohort by 35.50 units or by 79.78%, the highest improvement of all six statements. Self-evaluation scores according to participant demographics can be seen in Table 5.

**Table 5:** Self-evaluation scores for the statement “My capacity to respond appropriately if I suspect domestic violence.”

	**Pre-training**			**Post-training**		
	**n**	**Median**	**Mean**	**n**	**Median**	**Mean**
Total participants	36	44.50	44.14	19	80.00	81.95
**Gender identity**						
Female	33	42.00	42.85	14	76.50	79.79
Male	2	67.50	67.50	4	89.50	91.00
Other/ unspecified	1	40.00	40.00	1	76.00	76.00
**Age group (years)**						
18–25	6	33.50	32.50	3	70.00	72.67
26–35	13	40.00	42.38	3	80.00	80.33
36–45	9	50.00	48.33	5	78.00	83.00
46–55	7	50.00	51.13	7	89.00	87.00
56+	1	50.00	50.00	1	74.00	74.00
**Industry experience (years)**						
<5	18	36.00	40.44	7	80.00	83.29
5–10	6	45.00	52.33	3	76.00	78.33
11–20	7	50.00	46.43	5	90.00	87.00
21–30	3	50.00	37.33	3	75.00	76.67
31+	2	55.00	55.00	1	74.00	74.00
**Documented involvement in domestic violence programs**						
Yes	10	50.00	55.80	5	75.00	82.40
No	26	38.50	39.65	14	80.00	81.79
**Experienced personal threats of domestic violence**						
Yes	11	50.00	54.64	7	76.00	82.57
No	25	37.00	39.52	12	80.00	81.58

#### 3.1.5 Statement 5: “My capacity to respond appropriately to disclosures of domestic violence”

When asked to quantify “My capacity to respond appropriately to disclosures of domestic violence,” the median response in the pre-training survey was 50.00 (*n* = 36), while the median response in the post-training survey was 85.00 (*n* = 19). Thus, there was an overall increase in the median self-evaluation score of the cohort by 35.00 units or by 70.00%. Self-evaluation scores according to participant demographics can be seen in Table 6.

**Table 6:** Self-evaluation scores for the statement “My capacity to respond appropriately to disclosures of domestic violence.”

	**Pre-training**			**Post-training**		
	**n**	**Median**	**Mean**	**n**	**Median**	**Mean**
Total participants	36	50.00	46.89	19	85.00	82.53
**Gender identity**						
Female	33	50.00	46.45	14	80.00	80.14
Male	2	52.50	52.50	4	90.50	92.75
Other/ unspecified	1	50.00	1	50.00	75.00	75.00
**Age group (years)**						
18–25	6	41.50	37.00	3	75.00	76.33
26–35	13	50.00	48.46	3	85.00	83.80
36–45	9	40.00	42.78	5	85.00	83.80
46–55	7	62.00	59.43	7	90.00	85.14
56+	1	35.00	35.00	1	74.00	74.00
**Industry experience (years)**						
<5	18	50.00	48.72	7	86.00	84.71
5–10	6	50.00	54.17	3	75.00	78.67
11–20	7	30.00	34.00	5	90.00	87.80
21–30	3	62.00	51.00	3	68.00	75.33
31+	2	47.50	47.50	1	74.00	74.00
**Documented involvement in domestic violence programs**						
Yes	10	55.00	58.00	5	74.00	79.00
No	26	43.50	42.62	14	85.50	83.79
**Experienced personal threats of domestic violence**						
Yes	11	50.00	57.64	7	75.00	80.00
No	25	43.00	42.16	12	85.50	84.00

#### 3.1.6 Statement 6: “My capacity to refer appropriately, following disclosures of domestic violence”

When asked to quantify “My capacity to refer appropriately, following disclosures of domestic violence,” the median response in the pre-training survey was 48.00 (*n* = 36), while the median response in the post-training survey was 84.00 (*n* = 19). Thus, there was an overall increase in the median self-evaluation score of the cohort by 36.00 units or by 75.00%. Self-evaluation scores according to participant demographics can be seen in Table 7.

**Table 7:** Self-evaluation scores for the statement “My capacity to refer appropriately, following disclosures of domestic violence.”

	**Pre-training**			**Post-training**		
	**n**	**Median**	**Mean**	**n**	**Median**	**Mean**
Total participants	36	48.00	44.39	19	84.00	84.05
**Gender identity**						
Female	33	46.00	43.73	14	79.00	82.57
Male	2	52.50	52.50	4	90.50	91.25
Other/ unspecified	1	50.00	50.00	1	76.00	76.00
**Age group (years)**						
18–25	6	37.00	35.67	3	78.00	76.67
26–35	13	50.00	41.23	3	80.00	80.00
36–45	9	50.00	48.56	5	88.00	88.80
46–55	7	61.00	53.71	7	91.00	87.00
56+	1	35.00	35.00	1	74.00	74.00
**Industry experience (years)**						
<5	18	43.00	41.50	7	80.00	84.00
5–10	6	50.00	53.67	3	76.00	79.00
11–20	7	43.00	44.14	5	90.00	91.80
21–30	3	50.00	41.33	3	75.00	78.00
31+	2	48.00	48.00	1	74.00	74.00
**Documented involvement in domestic violence programs**						
Yes	10	55.50	56.70	5	86.00	84.00
No	26	43.00	39.65	14	82.00	84.07
**Experienced personal threats of domestic violence**						
Yes	11	50.00	55.27	7	86.00	83.71
No	25	40.00	39.60	12	82.00	84.25

### 3.2 Participant responses to pre- and post- training “calibrating” questions

Participants were asked a series of calibrating questions to give a deeper understanding of the impact of the training on their knowledge and understanding of key issues related to DV involving animals and animal abuse. The results, shown in Table 8, demonstrate that prior to the training, many participants already had a relatively good level of understanding of DV and animal abuse. Nevertheless, the training did improve on their existing knowledge on the topic. The biggest improvement was seen in participants' understanding of risk factors for DV. Prior to the training, only 55.6% of participants correctly identified gender as being the greatest risk factor for DV. Upon completion of the training, this increased to 100%. Interestingly, the mean score for participants' pre-training self-evaluation of their understanding of the gendered nature of DV was the highest of all pre-training self evaluation scores. The results from the calibrating question on gender, however, shows that participants' actual understanding of the gendered nature of DV may differ from their perceived understanding.

**Table 8:** Participant responses to pre- and post-training calibrating questions.

**Question**	**Correct answer**	**Pre-training correct (%)**	**Post-training correct (%)**	**% pt Change (+ or –)**
What is the greatest risk factor for those who experience domestic violence: (a) socioeconomic status; (b) culture; (c) religion; (d) gender; or (e) alcohol?	(d)	55.6	100	+44.4
A client mentions that their intimate partner forbids them from walking their dog with their friends. In the context of domestic violence this can be considered a form of abuse. What form of abuse would this be regarded as (select all that apply): (a) isolation; (b) physical abuse; (c) emotional/psychological abuse; (d) stalking (technology abuse)?	(a)	80.6	94.7	+14.1
Which of the following accurately reflects the responsibilities of a veterinary professional in responding to suspected pet abuse: (a) the veterinary professional has a professional responsibility to intervene but not a legal responsibility; (b) the veterinary professional has a legal responsibility to intervene regardless of their professional obligations; (c) the veterinary professional has both a professional and legal responsibility to intervene in suspected pet abuse; or (d) the veterinary professional has neither a professional nor a legal responsibility to intervene?	(a)	51.4	94.7	+43.3
Which of the following methods of approach would post the most amount of risk to a victim survivor of domestic violence in the veterinary clinical setting: (a) having posters and/or pamphlets from support services displayed privately in the bathrooms; (b) having posters and/or pamphlets from support services available publicly in the waiting room; (c) asking a question about the possibility of domestic violence in a respectful manner in the presence of the suspected perpetrator; or (d) asking a question about the possibility of domestic violence in a respectful manner after separating the suspected victim from the suspected perpetrator in a safe setting?	(c)	88.9	100	+ 11.1
True or false: a victim of domestic violence must have evidence of physical abuse to be eligible for support from specialist domestic violence service providers.	False	97.2	100	+2.8
Following disclosure of domestic violence by a client, which of the following FIRST responses would be most appropriate: (a) you provide counseling; (b) you provide case management support; (c) provide information on local specialist domestic violence service providers if safe and appropriate to do so; or (d) you offer to board their animal at your clinic?	(c)	94.4	100	+5.6
In Victoria, which of the following statements is true in situations of pet ownership disputes between victims and perpetrators of domestic violence: (a) the legal owner of the pet is dictated by who it lives with, regardless of microchip or council registration; (b) victims who apply for an Intervention Order are more likely to become the owners of a disputed pet; (c) in the family court system pets are considered “property,” meaning the presence of violence may not determine who gets to keep the pet; or (d) whoever pays for the pet's veterinary bills is legally considered the owner of the pet?	(c)	72.2	73.7	+1.5
**Question**	**Correct answer**	**Pre-training correct (mean)**	**Post-training correct (mean)**	**% pt Change (**+ **or –)**
What are the advantages of asking about a client's safety and wellbeing following disclosure or suspicion of domestic violence (select all that apply): (a) to overcome barriers to disclosing; (b) to show concern and encourage actions toward safety; (c) to ask them to make a decision right away; (d) to focus the conversation and give a victim “permission” to speak?	(a), (b), (d)	1.86	2.42	+30

The second largest change that the calibrating questions demonstrated was in participants' understanding of the responsibilities of veterinary professionals when responding to suspected pet abuse. Prior to the training, only 51.4% of participants were aware that veterinary professionals have a professional responsibility to intervene in pet abuse but not a legal responsibility. Following the training, this increased to 94.7%.

In all, the calibrating questions showed that participants' understanding of DV, animal abuse, and associated professional responsibilities improved following the training. Although some of the changes were small, the increases were consistent. These results support participants' self-evaluation that the training improved their capacity to recognize and respond to DV in a professional setting.

## 4 Discussion

The pre-training self-evaluation scores indicate that while most veterinary professionals are aware of the link between animal abuse and domestic violence and the gendered nature of DV, they lacked the capacity to respond and refer potential victims when confronted with suspicions or actual disclosures of abuse. This is reflected in the relatively higher baseline self-evaluation scores of statement 1 (64.50) and statement 2 (65.00) which are based on the knowledge and understanding, compared to the relatively lower self-evaluation scores of statement 3 (50.00), statement 4 (44.50), statement 5 (50.00), and statement 6 (48.00), which are based on the capacity to recognize, respond and refer. The baseline findings are consistent with the concerns previously identified by Newland, who highlighted the need for training in the recognition and identification of DV and the appropriate actions or relevant services to contact (35).

When comparing the self-evaluation survey results with the calibrating question results, some interesting discrepancies appear. Namely, that participants' existing knowledge on several key issues relating to animal abuse and DV was already relatively high prior to completing the training. However, participants' self-evaluated level of knowledge and capacity predominantly sat in the middle range for the six statements. What these results indicate is that participants may be underestimating their abilities when it comes to their level of knowledge and skills in correctly identifying and appropriately responding to suspected cases of DV and animal abuse. This underestimation is indicative of low confidence in their abilities, or low self-efficacy (62). And indeed, the results for pre-training self-evaluation survey questions did indicate that participants had low confidence in their ability to recognize, respond, and refer in cases of DV, as shown through the baseline self-evaluation scores of statements 3–6.

Veterinary professionals' lack of confidence in their abilities is likely to impact their responses to suspected cases of DV, as self-efficacy is a strong predictor of how well a person will perform in a given situation (62). Fortunately, following the training, participants' confidence levels improved, and they felt better equipped and prepared to respond when faced with suspected DV. With existing literature showing that many veterinary professionals report feeling uncertain and underprepared to respond to cases of DV involving animals (35, 45, 52), the impact of this training in removing uncertainty is promising.

Another issue that the results highlighted was the need for wider referral networks and procedures for DV victim-survivors who present at veterinary clinics. With a pre-training median self-evaluation score of 48.00 for Statement 6, many veterinary professionals lacked confidence and capacity to refer potential victims of DV, suggesting that the veterinary professionals involved in the study would benefit from standardized protocols for responses. Collaboration with local DV services would assist veterinary practices to determine the safest and most appropriate route of referral for their area, as this can vary widely across regions.

Of the 39 participants who completed the pre-training survey, only 17 proceeded to complete the post-training survey. This resulted in an attrition rate of 43.59%, leaving the study open to attrition bias. It is also important to highlight again that this study is exploratory, and that due to the small sample size, the results are indicative only. However, the results are nonetheless useful to contribute to the evidence base of what may work to facilitate veterinary professionals' capacity to recognize and respond to DV. What the post-training survey results do say about the effectiveness of the pilot program on this cohort of participants is that in every focus area, training improved veterinary professionals' self-reported ability to recognize, respond, and refer human DV victim-survivors, with these results supported by the consistent increases in correct response rates to the calibrating questions. The most significant improvement could be seen in participants' self-reported ability to respond appropriately if they suspected DV. In the pre-training survey, the median response was 44.5 (*n* = 36), while in the post-training survey, the median score was 80.00 (*n* = 19), an overall increase in the median score of 35.5 units or by 79.78%. For this cohort of Vet 3-R's training program participants, the program was effective in increasing self-identified levels of understanding of how DV and animal abuse can intersect, and how to support their clients who may be or who are experiencing abuse.

## 5 Conclusion

This study aimed to measure the impact of one pilot training program on veterinary professionals' ability to recognize, respond and refer in cases of known or suspected DV. Responses from participant surveys suggest that the EDVOS Vet-3R's training resulted in an increase in respondents' understanding of the link between DV and animal abuse, and the gendered nature of DV. This increase was also seen in participants' self-evaluated ability to translate this knowledge increase into practice through recognizing, responding, and referring their clients to specialist support if required. Although this research is based on a small sample of training participants, it nevertheless provides an indication that the Vet-3R program can be an effective tool for equipping veterinary professionals to safely assist DV victim-survivors. More research on similar programs with a larger cohort of participants would be beneficial to measure the impact of such programs on a wider scale. These findings beg the question: are veterinary professionals an underutilized asset in domestic violence prevention and response measures, and is there potential for vets to be trained to act as an additional intervention point for DV victim-survivors with animals?

## Data availability statement

The raw data supporting the conclusions of this article will be made available by the authors, without undue reservation.

## Ethics statement

The studies involving humans were approved by University of Melbourne office of research ethics and integrity. The studies were conducted in accordance with the local legislation and institutional requirements. The participants provided their written informed consent to participate in this study.

## Author contributions

RP: Writing—original draft, Formal analysis, Investigation. EB: Writing—review & editing, Conceptualization, Formal analysis, Investigation, Methodology, Supervision, Validation, Writing—original draft. YK: Writing—review & editing, Funding acquisition, Project administration, Resources. KH: Writing—original draft, Writing—review & editing. KD: Resources, Supervision, Writing—review & editing.

## Footnotes

^1^EDVOS has restructured into a different DV services now called Family Violence Free (FVREE). Vet-3R's training is now being offered as “Animal-3R's” training.
